# Conjunctival Intraepithelial Lymphocytes, Lacrimal Cytokines and Ocular Commensal Microbiota: Analysis of the Three Main Players in Allergic Conjunctivitis

**DOI:** 10.3389/fimmu.2022.911022

**Published:** 2022-07-19

**Authors:** José Carlos Zarzuela, Roberto Reinoso, Alicia Armentia, Amalia Enríquez-de-Salamanca, Alfredo Corell

**Affiliations:** ^1^ Department of Immunology, University of Valladolid, Valladolid, Spain; ^2^ Ocular Surface Group, Institute for Applied Ophthalmobiology (IOBA), University of Valladolid, Valladolid, Spain; ^3^ Department of Allergy, Hospital Universitario Río Hortega, University of Valladolid, Valladolid, Spain; ^4^ Biomedical Research Networking Center in Bioengineering, Biomaterials and Nanomedicine (CIBER-BBN), Madrid, Spain

**Keywords:** conjunctiva, IELs, tear, cytokines, microbiota, allergic conjunctivitis

## Abstract

Conjunctival intraepithelial lymphocytes, tear soluble molecules and commensal microbiota have important roles in the ocular mucosal immune response in healthy and diseased subjects. For the purpose of this study, the cellular and microbial populations of the conjunctiva and the lacrimal soluble molecules were analyzed to find the main biomarkers in allergic conjunctivitis. A total of 35 healthy subjects, 28 subjects with seasonal allergic conjunctivitis and 32 subjects with perennial allergic conjunctivitis were recruited to obtain peripheral blood, conjunctival brush cytology, tear fluid and microbiota samples. Flow cytometry for lymphocytes, multiplex bead assays for cytokines and high-throughput DNA sequencing for microbiome analysis were used. For perennial allergic conjunctivitis, an increased proportion of Th2 and NKT lymphocytes was found, while CD3+TCRγδ+ lymphocytes and double negative MAIT cells were decreased. In contrast, seasonal allergic conjunctivitis was distinguished by an increase in Th17 and Th22 cell proportions, while the Th1 cell proportion decreased. Among tear fluid, the vast majority of pro-inflammatory cytokines (especially Th2 and Th17 cytokines) in perennial allergies and MMP-9 together with IgA in seasonal allergies were increased. In contrast, TGF-β2 was decreased in both forms of conjunctivitis. Finally, fungal (*Malassezia* species) and bacterial (*Kocuria* and *Propionobacterium acnes* species) colonization were observed in the perennial allergic conjunctivitis group. These results provide the basis for the development of a disease profile for perennial allergic conjunctivitis and open the door to new therapeutic and diagnostic strategies.

## Introduction

The conjunctiva and tears constitute the main defensive barrier against pathogens on the ocular surface. The conjunctiva, like other mucous membranes, has an associated lymphoid tissue (CALT) that protects the ocular surface from numerous infections. the in-depth functions of CALT, its relationship with the lacrimal component and which factors are involved in the allergic process in this tissue have yet to be elucidated.

It is known that the ocular surface will react and activate the immune system when an allergen comes into contact with it, triggering a mild (healthy conjunctiva) or hypersensitive (allergic conjunctiva) response. Allergic diseases are one of the main reasons for medical consultation. The prevalence of allergic diseases is dramatically increasing and is expected to continue to grow in the coming years. The prevalence of allergic conjunctivitis is currently around 30-40% (1, 2). There are several types of allergic conjunctivitis: vernal and atopic keratoconjunctivitis (chronic) and perennial and seasonal allergic conjunctivitis (acute), with the last two being the most common types. All of them may have similar symptoms (itching, redness, and tearing), but the immunological process/immune response, both cellular and humoral, could be completely different.

Mucosa-associated lymphoid tissue has been studied for years, especially the gastric mucosa and its immune system’s role in food allergy or intolerance. In contrast, the immune system of the eye, and particularly the mechanisms involved in the defense of the ocular surface, has yet to be discovered. It is important to note how the pathogen response will be determined by the interactions of many elements, including molecular (cytokines, chemokines, enzymes, immunoglobulins, etc.), cellular (epithelium, lymphocytes, monocytes, goblet cells, etc.) and microbial (commensal fungi and bacteria).

To date, various studies have been performed to identify the tear inflammatory mediators in various ocular conditions. They have confirmed that there is great variability in the pro-inflammatory cytokine concentrations in some pathologies. Cytokines, such as IL-2, IL-4, IL-5, IL-13, IFN-γ, RANTES and eotaxin, are closely related to symptomatology in the different types of ocular allergy (3–5). This is mainly due to the work of conjunctival lELs, which are responsible for mediating the response and release of pro-inflammatory molecules into tears. NK, NKT and Th lymphocytes (Th1, Th2 and Th17) actively contribute to the composition of tears, which will affect the type of humoral response produced and may even aggravate the patient’s symptomatology. Therefore, it seems reasonable to think that, as occurs in other mucosal areas, there is some variability in the phenotypes of conjunctival intraepithelial lymphocytes (IELs), depending on allergy etiology.

Although the molecular component of tears is now widely studied in allergy, a detailed characterization of the different populations and subpopulations of conjunctival IELs is still needed. As previously reported (6), the upper tarsal conjunctiva constitutes one of the major areas of immune activity and IEL infiltration, with a predominance of CD8+ vs CD4+ T lymphocytes.

At present, there is controversy, and it is unclear who plays a stronger role in the ocular allergic response. Many authors agree that the major mediator of the immune response to ocular allergy is the Th2 lymphocyte together with the Th17 lymphocyte (7, 8). Other authors suggest that innate immune cells (mainly NKT, TCRγδ lymphocytes and mast cells) play an important role in ocular surface defense (9).

Finally, the other main player emerging in recent years is the resident microbiota and its contribution to inflammatory response (10). Some authors report the existence of a kind of symbiosis between commensal microbiota, lymphocytes and epithelial cells (11) and suggest that the allergic response may be influenced by an ocular surface dysbiosis (microbial imbalance) affecting their physiology and integrity (12).

Although the tear in different ocular pathologies is currently widely studied, no previous studies have analyzed and interlinked the three main factors involved in the ocular surface inflammatory response: IELs, lacrimal molecules and commensal microbiota.

With this study, we aim to provide an overview of the mechanisms involved in ocular defense. For this purpose, we will compare the regional (upper tarsal conjunctiva and tears) with the circulating (peripheral blood) immune system; make a detailed characterization of the conjunctival IELs, lacrimal soluble molecules and commensal microbiota of healthy individuals; and identify the differences with patients who have allergic conjunctivitis. Finally, we will analyze the obtained data to find differential biomarkers to improve the diagnosis and treatment of allergic conjunctivitis.

## Materials and Methods

### Subjects

A total of 95 subjects (35 healthy and 60 allergic) in the age range of 10 to 76 years were recruited. From them, different samples of peripheral blood and plasma were obtained by venipuncture of the arm, tears by capillary and conjunctival cells by brush cytology. The pre-diagnosed (via prick test or specific IgE determination) allergic patients were subdivided according to their allergy etiology: 28 with seasonal allergic conjunctivitis (SAC) and 32 with perennial allergic conjunctivitis (PAC). Conjunctivitis was diagnosed by a professional ophthalmologist. Allergic symptoms such as itching, redness, tearing, discharge, papillae, inflammation, sneezing or other nasal symptoms were used to differentiate allergic from non-allergic conjunctivitis. Allergies to grass pollen: bermuda grass (28.6%) and timothy grass (42.8%); hay (3.6%); and tree pollen: olive (10.7%), cypress (7.1%), plane tree (3.6%), and Japanese cedar (3.6%) were included in the SAC group. While allergies to mite (35.3%), cat (35.3%), dog (26.5%), and rabbit (2.9%) were included in the PAC group. A total of 80 peripheral blood and plasma samples, 73 conjunctival cytology samples, 81 tear samples and 22 microbiota samples were collected.

Patients diagnosed with allergy to pollens, dust/mites or animals by prick test or specific IgE test and allergic conjunctivitis were included in the allergy groups.

In contrast, patients suffering from chronic autoimmune or autoinflammatory pathologies, hematological neoplasms, hematopoietic progenitor transplants, radiotherapy or chemotherapy; who had undergone systemic or topical immunosuppressive, immunomodulatory or anti-inflammatory pharmacological treatment (excluding antihistamines) in the previous 6 weeks; acute inflammatory conditions of non-allergic origin in the previous 2 weeks; had undergone any type of previous ocular surgery; or long-term contact lenses wear in the previous 2 weeks were excluded.

In addition, healthy individuals who did not have a normal OSDI (Ocular Surface Disease Index), TBUT, fluorescein or Schirmer’s test were also excluded.

Informed consent was obtained from all the study subjects prior to sample collection. The study was conducted in accordance with the tenets of the Declaration of Helsinki.

The study protocols were approved by the clinical research ethics committee of each collaborating Public Health Center. Healthy volunteers were recruited through mass emailing by the University of Valladolid, while patients diagnosed with allergies and conjunctivitis were referred by Hospital Clínico Universitario, Hospital Rio Hortega, Centro de Salud Pilarica/Circular and IOBA of Valladolid.

Data about the number of samples, age, gender, questionnaire, OSDI, and ocular surface evaluation tests are summarized in **Table 1**.

**Table 1:** Information about number of subjects, age, sex, number of samples and relevant data obtained from the questionnaire used and the ocular examination.

	SAC	PAC	Control
No. of subjects	28	32	35
Mean age (years)	31.39 ± 18.15	34.87 ± 14.79	45.71 ± 16.57
Age range	10 to 66	13 to 67	19 to 76
Sex (Male : Female)	18:10	10:22	17:18
**No. of samples**			
Peripheral blood samples	22	29	29
Brush citology samples	23	26	24
Tear samples	23	29	29
Microbiome samples	6	9	7
**Lifestyle habits**			
Habitual residence (urban)	93%	90%	57%
Alcohol (at least once a week)	46%	70%	77%
Smoker	11%	21%	14%
Physical activity (2 to 4 times per week)	93%	65%	66%
Sleeping hours per day (less than 8 hours)	68%	78%	77%
Dietary supplement intake	7%	25%	14%
**History of diseases**			
Asthma	28%	25%	0%
Rhinitis	39%	34%	14%
Atopic dermatitis	39%	37%	6%
Bronchitis	11%	16%	6%
Conjuntivitis	75%	3%	17%
**In the last three months**			
Oral antihistamines	39%	16%	3%
Ocular antihistamines	28%	28%	8%
Artificial tears use	14%	6%	26%
Contact lens wear	25%	4%	6%
**Ocular tests**			
OSDI average	5	4	4
TBUT	>10	>10	>10
Conjunctival papillae (Grade I)	28%	37%	28%
Conjunctival papillae (Grade II)	21%	19%	6%
Fluorescein staining (Grade I)	14%	40%	26%
SCHIRMER test average	12mm	14mm	13mm

It was a prerequisite that all subjects had not used: antibiotics for at least 15 days, antihistamines for at least 48-72 hours and contact lenses for at least 7 days.

### Procedure

All patients were assessed using the procedure detailed below:

1) Questionnaire on lifestyle habits, general health status and ophthalmological history, 2) OSDI) test, 3) tear collection, 4) fluorescein TBUT, 5) tarsal conjunctival papillae evaluation, 6) corneal and conjunctival fluorescein evaluation according to the Oxford scale, 7) ocular physiological saline solution washing, 8) topical anesthetic instillation (1 mg/mL tetracaine hydrochloride + 4 mg/mL oxybuprocaine hydrochloride), 9) Schirmer’s test, 10) upper tarsal brush cytology (only in one eye), 11) lower bulbar conjunctival swab (in the other eye), and 12) peripheral blood collection.

### Sample Collection

Tarsal conjunctival cell samples were collected by brush cytology (BC) over the everted upper eyelid using conjunctival brushing technique based on previous reports (6). Cells were resuspended by gentle rotation for 30 s in an Eppendorf tube containing 1.2 mL of culture medium (supplemented RPMI-1640 medium with 10% FBS, 1% penicillin/streptomycin and 1% L-glutamine). The procedure was repeated three times to obtain a sufficient number of cells. Each tube was washed with 2 mL of cell wash solution and centrifuged to keep the cellular part. Peripheral blood samples were extracted by venipuncture and collected in EDTA tubes.

Tear collection was performed before any other tests. Unstimulated tear samples were collected non-traumatically from the external canthus of open eyes, avoiding additional tear reflexes as much as possible; 4 μL glass capillary micropipettes (Drummond, Broomall, PA) were used to collect 8 μL of tears from each eye. Tubes with tear samples were kept cold (4°C) during collection.

Cell samples were stained and analyzed within 24 hours, while plasma and tear samples were stored at -80°C for further analysis.

Finally, the microbiota sample was extracted by swabbing the lower bulbar conjunctiva and immediate collection in a clean tube filled with 1 mL of phosphate-buffered saline (PBS) and sent for molecular analysis to the Biome Makers Laboratory in Valladolid.

### Sample Analysis

#### Flow Cytometry

Blood and conjunctival samples were analyzed by flow cytometry with a Cytomics FC 500 cytometer (Beckman Coulter, Fullerton, CA). The data collected were analyzed using the Cytomics RXP software program (Beckman Coulter).

Controls included the cross reactivity of the fluorescence signals of each channel and the isotype-matched unspecific monoclonal antibodies used as negative controls. Conjunctival and blood cell phenotypes were determined by conjugated mouse anti-human monoclonal antibodies staining. Five antibody panels were configured, as shown in **Table 2**.

**Table 2:** Description of monoclonal antibodies used.

	Cell Marker	Color	Clone	Isotype	Manufact	Panel
**Reactivity: Mouse Anti-Human**	**CCR10**	APC	6588-5	IgG	BioLegend	2
	**CD3**	APC - Alexa 750	UCHT1	IgG1, K	Beckman Coulter	1, 3, 4, 5
	**CD4**	ECD	SFCI12T4D11	IgG1	Beckman Coulter	2
	**CD4**	APC	13B8.2	IgG1	Beckman Coulter	1, 3
	**CD5**	APC	L17F12	IgG2a	Immunostep	5
	**CD8**	PE	3B5	IgG2a	Caltag	4
	**CD8**	ECD	SFCI21Thy2D3	IgG1	Beckman Coulter	1
	**CD16**	FITC	3G8	IgG1	Immunostep	4
	**CD19**	FITC	4G7	IgG1, K	Becton Dickinson	5
	**CD25**	PE	BC96	IgG1, K	BioLegend	3
	**CD45**	ECD	J33	IgG1	Beckman Coulter	3, 4, 5
	**CD45R0**	PE	UCHL1	IgG2a, K	Becton Dickinson	1
	**CD45RA**	FITC	L48	IgG1, K	Becton Dickinson	1
	**CD56**	APC	MEM-188	IgG2a, K	Immunostep	4
	**CD127**	FITC	R34.34	IgG1	Beckman Coulter	3
	**CD183**	FITC	G025H7	IgG1, K	BioLegend	2
	**CD194**	PE/Cy7	L291H4	IgG1, K	BioLegend	2
	**CD196**	PE	G034E3	IgG2b, K	BioLegend	2
	**TCR pan γ/δ**	PE	IMMU510	IgG1	Beckman Coulter	5

The manufacturer’s recommended amounts were used for peripheral blood, while only half was used for conjunctival samples. APC= allophycocyanin. ECD= phycoerythrin-Texas Red-X. FITC= fluorescein isothiocyanate. PE= phycoerythrin. PE/Cy7= phycoerythrin-cyanine 7.

Peripheral blood (250 μL) and conjunctival (1.2 mL) samples were divided equally into five different tubes. Conjunctival cells were washed with 2 mL of cell wash solution (BD Biosciences, San Jose, CA) and centrifuged at 500 × g for 5 min. Then, both samples were stained with conjugated mouse anti-human monoclonal antibodies and incubated in the dark at room temperature for 15 min. Samples were then incubated in the dark with 0.5 mL of FACS lysing solution (BD Biosciences) at room temperature for 15 min to lyse any residual red cells under gently hypotonic conditions and also to preserve epithelial cells and leukocytes. Afterwards, the cells were gently agitated, and flow cytometry analysis was performed. The data collected were analyzed using the Cytomics RXP software program and Cytomics FC 500 cytometer (Beckman Coulter, Fullerton, CA). Gating procedures are summarized in **Supplementary Figure 1**

Controls included the cross reactivity of the fluorescence signals of each channel and isotype-matched unspecific monoclonal antibodies used as negative controls. The cytometer was adjusted for a 400 s sample acquisition time for conjunctival samples and 200 s for peripheral blood. An average of 100,000 events in peripheral blood and 20,000 events in the conjunctiva were counted per tube.

#### Cytokine/Chemokine and Ig Determination (Luminex Assay)

Tear and plasma samples were analyzed with Luminex IS-100 equipment (Luminex Corporation, Austin, TX, USA) using commercial bead-based arrays according to the manufacturer’s instructions. Six Milliplex multiplex assays (from Merck-Millipore, Millipore Iberica, Madrid, Spain) were used for the analysis of the following soluble molecules (minimum detectable concentrations in pg/mL for each soluble molecule in brackets):

Custom HCYTOMAG-60K: Eotaxin (3.08), IFN-γ (0.86), IL-1β (0.52), IL-2 (0.28), IL-4 (0.20), IL-5 (0.17), IL-6 (0.14), IL-8 (0.52), IL-9 (2.20), IL-10 (0.91), IL-12P40 (3.24), IL-12P70 (0.88), IL-13 (2.58), IL-17A (0.71), IL-17E (6.00), IL-17F (28.63), IL-21 (2.00), IL-22 (12.68), MCP-1 (3.05), RANTES (1.58), TNF-α (5.39). HMMP2MAG-55K: MMP-9 (2.00). TGFBMAG-64K-03: TGF-β1 (6.00), TGF-β2 (6.60) and TGF-β3 (2.20). HCYP2MAG-62K: TSLP (3.1). HGAMMAG-301K: IgA (400). HGAMMAG-303E: IgE (400).

Briefly, 50 μL of plasma and 10 μL of the 1:10 diluted tear sample were incubated under agitation (from 1 h to overnight incubation, according to protocol) at 4°C with beads. Then, samples were first incubated for 1 h with biotinylated detection antibodies followed by incubation with streptavidin-phycoerythrin for 30 min. For TGF-β multiplex assays, samples were previously acidified adding 2.0 μL of 1.0 N hydrochloric acid to each 25 μL of the diluted sample, following manufacturer instructions. Standard curves were used to convert fluorescence units to concentration units (pg/mL). Data was stored and analyzed with “Bead View Software” (Upstate-Millipore Corporation, Watford, UK).

#### Microbiome High-Throughput DNA Sequencing

Samples were immediately sent for molecular analysis to the Biome Makers Laboratory in Valladolid. DNA extraction was performed with the DNeasy PowerLyzer PowerSoil Kit from Qiagen. To characterize both bacterial and fungal microbial communities associated with the samples, the 16S rRNA and internal transcribed spacer (ITS) marker regions were selected. Libraries were prepared following the two-step PCR Illumina protocol using custom primers amplifying the 16S rRNA V4 region and the ITS1 region. Sequencing was conducted in an Illumina MiSeq instrument using pair-end sequencing (2 × 300 bp).

#### Statistical Analysis

Statistical analysis was carried out using IBM SPSS version 24.0.0.2. All obtained data were expressed as means ± SDs. For cytokines, levels of all molecules were analyzed as base 2 log-transformed variables. Normality assumption was checked by the Shapiro-Wilk test and homogeneity of variances by the Levene test. The Student’s t-test or the non-parametric Mann-Whitney U test (if normality could not be assumed), applying the Bonferroni correction was used to compare groups. Correlations between the peripheral blood and upper tarsal conjunctiva flow cytometry data were determined by Spearman’s rho correlation coefficient. Single symbols for p < 0.05, double symbols for p < 0.01 or triple symbols for p < 0.001 were used to indicate the significance range. Age and sex differences were analyzed to discard any existing statistical influence. Statistical analyses of microbiota data were done mainly using phyloseq and microbiome R packages (13, 14). Microbiome analyses were carried out using R programming. Alpha diversity was computed based on Shannon Index for 16S and ITS rarefied reads. Analysis of variance of pathology categories per sex was determined using Kruskall-Wallis test. Beta diversity was calculated on compositional data using Principal Coordinate Analysis (PCoA) ordination and Bray-Curtis distance matrix.

### Results

#### Healthy Conjunctiva Characterization. Main Differences with the Peripheral Immune System

Using flow cytometry, we have been able to characterize conjunctival IELs and analyze the main differences with the peripheral blood immune system. The values obtained in healthy individuals in both tissues are summarized in **Table 3**.

**Table 3:** Lymphoid subset proportions in peripheral blood (PB) and upper tarsal conjunctiva (UTC) in healthy individuals.

Lymphoid subtype	Cell markers	PB (%)n = 29	UTC (%)n = 24	P-value
**T cells**	CD3^+^	76.63 ± 4.95	80.48 ± 8.53	*
**dn MAIT cells**	CD3^+^CD8^-^CD4^-^	5.28 ± 4.67	16.33 ± 9.82	***
**TCRγδ^+^ cells**	CD3^+^TCRγδ^+^	2.11 ± 1.62	33.89 ± 14.22	***
**CD4^+^ T cells**	CD3^+^CD4^+^	47.26 ± 8.69	28.03 ± 9.98	***
**CD4^+^ naïve T cells (Th0)**	CD3^+^CD4^+^CD45RA^+^	27.82 ± 7.29	13.87 ± 7.48	***
**CD4^+^ memory T cells**	CD3^+^CD4^+^CD45R0^+^	25.72 ± 8.57	22.48 ± 9.33	
**Treg cells**	CD3^+^CD4^+^CD25^hi^CD127^lo^	2.27 ± 1.00	8.78 ± 4.37	**
**Th1**	CD4^+^ CD183^+^CD194^-^CD196^-^CCR10^-^	8.75 ± 7.49	3.78 ± 3.25	***
**Th17/Th1**	CD4^+^ CD183^+^CD194^-^CD196^+^CCR10^-^	4.51 ± 2.58	1.78 ± 1.84	***
**Th2**	CD4^+^ CD183^-^CD194^+^CD196^-^CCR10^-^	3.43 ± 3.28	1.46 ± 1.76	*
**Th17**	CD4^+^ CD183^-^CD194^+^CD196^+^CCR10^-^	2.79 ± 2.43	1.56 ± 1.87	
**Th22**	CD4^+^ CD183^-^CD194^+^CD196^+^CCR10^+^	1.32 ± 1.60	1.08 ± 1.36	
**CD8^+^ T cells**	CD3^+^CD8^+^	24.72 ± 9.02	36.12 ± 14.39	***
**CD8^+^ naïve T cells**	CD3^+^CD8^+^ CD45RA^+^	17.25 ± 8.18	28.4 ± 14.32	**
**CD8^+^ memory T cells**	CD3^+^CD8^+^ CD45R0^+^	13.72 ± 6.79	24.91 ± 12.46	**
**CD8^+^NKT CD56^+^CD16^-^**	CD3^+^CD8^+^ CD56^+^CD16^-^	4.38 ± 7.05	5.33 ± 5.83	
**CD8^+^NKT CD56^+^CD16^+^**	CD3^+^CD8^+^ CD56^+^CD16^+^	0.08 ± 0.07	6.17 ± 6.32	***
**CD8^+^NKT CD56^-^CD16^+^**	CD3^+^CD8^+^ CD56^-^ CD16^+^	–	0.96 ± 1.15	
**Total CD8^+^NKT**		4.46 ± 7.08	12.46 ± 8.76	***
**NK CD56^+^CD16^-^**	CD3^-^ CD56^+^CD16^-^	2.69 ± 2.86	4.10 ± 5.97	
**NK CD56^+^CD16^+^**	CD3^-^ CD56^+^CD16^+^	5.23 ± 3.74	1.39 ± 1.91	***
**NK CD56^-^CD16^+^**	CD3^-^ CD56^-^CD16^+^	5.35 ± 3.37	7.07 ± 8.71	
**Total NK**		13.27 ± 4.78	12.56 ± 9.96	
**CD19^+^ B cells**	CD19^+^	11.69 ± 3.52	6.71 ± 4.51	***
**B_1_ cells**	CD19^+^ CD5^+^	4.29 ± 3.78	4.76 ± 3.35	
**B_2_ cells**	CD19^+^ CD5^-^	7.40 ± 3.07	1.95 ± 2.48	***

Results calculated on the total leukocyte population (CD45+) and expressed as means ± SDs and percentages (%). dn MAIT cells = double negative mucosal associated invariant T cells. TCR = T cell receptor. Treg = regulatory T cells. Th= helper T cells. NKT= natural killer T cells. NK= natural killer cells. *p < 0.05; **p < 0.01; ***p < 0.001.

Several differences between healthy peripheral blood and conjunctiva have been found. High values of TCRγδ+ lymphocytes (33.89 ± 14.22%) and double negative mucosal associated invariant T cells (dn MAIT: 16.33 ± 9.82%) have been observed in the conjunctiva.

In peripheral blood, CD4 T lymphocytes represent a higher percentage (CD4: 47.26 ± 8.69% vs CD8: 24.72 ± 9.02%) of T cells, whereas a CD8 T lymphocyte predominance was observed in healthy conjunctiva (CD4: 28.03 ± 9.98% vs CD8: 36.12 ± 14.39%). Moreover, the polarization of CD4+ lymphocytes towards memory lymphocytes is stronger in the conjunctiva, where CD4+CD45R0+ T cells are almost twice as frequent as CD4+CD45RA+ T cells. Within CD4+ T cell subtypes, Treg cells (8.78 ± 4.37%) are the most common in the conjunctiva, while Th1 is the predominant subtype in peripheral blood (8.75 ± 7.49%).

Among CD8+ subsets, CD8+NKT cells have higher percentages in the conjunctiva (UTC: 12.46 ± 8.76% vs PB: 4.46 ± 7.08%). Regarding NK lymphocytes, although total NK lymphocytes had similar ratios in both samples, a decrease in NK CD56+CD16+ cells was observed in the conjunctiva, so lower cytotoxic activity was involved.

Finally, we found that total B lymphocytes (CD19+) were decreased in the conjunctiva, with B2 lymphocytes being responsible for this decrease. Interestingly, the predominance of B1 over B2 cells found in the conjunctiva was not observed in peripheral blood. B1 cells are involved in innate and humoral immunity, producing high quantities of immunoglobulin, as expected.

It is important to note that all these differences between the blood and conjunctiva have also been observed in both perennial and seasonal allergic conjunctivitis, suggesting that they are pathology-independent differences (data not shown).

#### Correlation of the Regional (Upper Tarsal Conjunctiva) and Circulating (Peripheral Blood) Immune System

Comparing the proportions of IELs and peripheral blood lymphocytes, a correlation between the regional and circulating immune systems was found in some lymphocyte populations.

Moderate positive correlations were found for Th22, total NKT, naïve CD8 T (CD3+CD8+CD45RA+) and memory (CD3+CD8+CD45R0+) T cells, as shown in **Supplementary Figure 2**. In contrast, when comparing plasma and tear inflammatory molecule concentrations, no correlation was found.

### Conjunctival Immune Alterations in Allergic Subjects

#### Lymphoid Populations

**Figure 1:**
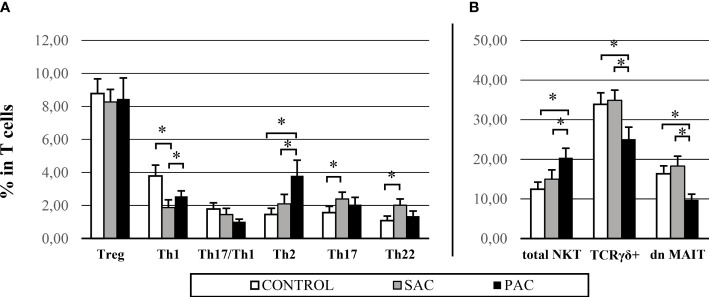
Conjunctival IEL proportions – main differences in control, SAC and PAC groups. Graphs are divided into: CD4+ populations **(A)** and innate cells **(B)**. NKT = natural killer T cells. TCR γδ+ = gamma delta T cell receptor. dn MAIT= double negative mucosal associated invariant T cells. *p > 0.05.

Comparing healthy and allergic conjunctiva, statistically relevant results were found, as can be seen in **Figure 1**. A decrease in Th1 cell proportions was detected in allergic conjunctiva, which was more pronounced in seasonal allergies (CONTROL: 3.78 ± 3.25% vs SAC: 1.87 ± 2.21% vs PAC: 2.56 ± 1.58%). In contrast, the Th2 subset in PAC (1.46 ± 1.76% vs 3.80 ± 4.51%) and Th17 (1.56 ± 1.87% vs 2.39 ± 2.03%) and Th22 (1.08 ± 1.36% vs 202 ± 1.84%) subsets in SAC were significantly increased (**Figure 1A**).

Among innate populations, an increase in total NKT proportion in PAC (C: 12.46 ± 8.76% vs SAC: 14.98 ± 11.53% vs PAC: 20.42 ± 12.16%) was observed. In contrast, TCRγδ+ (C: 33.89 ± 14.22% vs SAC: 34.88 ± 12.70% vs PAC: 25.10 ± 15.36%) and dn MAIT cells (C: 16.33 ± 9.82% vs SAC: 18.29 ± 12.30% vs PAC: 9.77 ± 7.39%) were strongly decreased compared to the SAC and control groups (**Figure 1B**).

#### Lacrimal Inflammatory Molecules

Several statistically significant differences were found for most of the analyzed molecules. In general terms, perennial conjunctivitis had the highest concentrations in almost all cases. Seasonal allergies, by contrast, showed concentrations similar to the healthy group. Results are summarized in **Table 4**.

**Table 4:** Obtained concentrations for soluble molecules analyzed in tears.

	Control	Seasonal	Perennial			
Number	29	23	29			
Sex (male:female)	13:16	16:07	8:21			
Age (years)	46.69 ± 15.75	33.61 ± 17.00	35.93 ± 14.89			
**Molecule (pg/mL)**	**Mean ± SD**	**Mean ± SD**	**Mean ± SD**	**CvsS**	**CvsP**	**SvsP**
EOTAXIN	6.82 ± 4.89	6.67 ± 4.26	22.21 ± 50.73			
IFN-γ	19.87 ± 25.89	15.31 ± 19.19	44.44 ± 62.53		♯♯	***
IgA	42641.19 ± 23165.67	63883.20 ± 54198.03	39877.52 ± 40230.88			*
IgE	82748.40 ± 80463.55	134511.94 ± 107468.61	191803.86 ± 163114.95	+	♯♯	
IL-1β	3.03 ± 6.77	1.61 ± 2.71	11.05 ± 25.42		♯	**
IL-2	10.77 ± 15.37	9.22 ± 9.32	23.67 ± 41.79		♯♯	*
IL-4	238.72 ± 380.28	250.77 ± 349.60	640.06 ± 994.80		♯	
IL-5	11.91 ± 13.67	12.39 ± 11.05	26.87 ± 31.27		♯♯	*
IL-6	16.09 ± 18.96	11.68 ± 15.58	29.75 ± 38.28			*
IL-8	101.37 ± 124.41	71.31 ± 105.56	84.17 ± 132.18			
IL-9	2.44 ± 1.82	2.64 ± 2.24	4.10 ± 5.12			
IL-10	15.51 ± 23.01	10.52 ± 14.98	33.02 ± 50.69			*
IL-12p40	6.82 ± 16	4.17 ± 10.32	19.79 ± 42.61			
IL-12p70	36.23 ± 47.5	25.91 ± 38.73	71.53 ± 109.77			*
IL-13	31.67 ± 42.09	37.18 ± 38.32	71.93 ± 95.22		♯	
IL-17A	4.71 ± 11.77	3.24 ± 10.48	12.78 ± 26.55			*
IL-17E	8.00 ± 5.26	8.38 ± 5.32	18.36 ± 25.14		♯♯♯	*
IL-17F	53.08 ± 63.18	58.32 ± 65.25	113.64 ± 142.93		♯	
IL-21	23.64 ± 46.77	24.61 ± 40.94	67.55 ± 107.11		♯	
IL-22	50.93 ± 93.54	54.21 ± 99.14	147.21 ± 262.62		♯	
MCP-1	576.35 ± 831.06	362.39 ± 303.33	308.12 ± 310.65			
MMP-9	4607.26 ± 6180.49	9258.95 ± 21911.86	4270.97 ± 11367,52			*
RANTES	71.93 ± 69.46	54.52 ± 56.35	118.66 ± 130.17			*
TGF-β_1_	8.81 ± 1.21	8.57 ± 1.26	8.54 ± 0.72			
TGF-β_2_	9532.22 ± 4663.8	6430.5 ± 2995.12	5548.88 ± 3480.88	+	♯♯♯	
TGF-β_3_	10.11 ± 3.76	9.92 ± 5.65	8.83 ± 1.37			
TNF-α	9.87 ± 13.46	6.64 ± 10.22	20.57 ± 29.74			*
TSLP	28.59 ± 27.12	27.72 ± 17.68	38.01 ± 16.43		♯	*

C = control, S = seasonal allergic conjunctivitis. P = perennial allergic conjunctivitis. IFN = interferon. Ig = immunoglobulin. IL = interleukin. MCP-1 = monocyte chemoattractant protein-1. MMP- 9= matrix metalloproteinase 9. TGF = transforming growth factor. TNF = tumor necrosis factor. TSLP = thymic stromal lymphopoietin. The non-parametric Mann-Whitney U test was used for statistical analysis. *, ♯, + : p < 0.05; **, ♯♯, ++ : p < 0.01; ***, ♯♯♯ and +++ : p < 0.001.

Comparing the control and allergic groups, altered concentrations of a huge collection of proteins were found. As expected, decreased TGF-β2 and increased IgE concentrations were observed in both allergic groups. In contrast, concentrations of other molecules were significantly increased in PAC, including IFN-γ, IL-1β, IL-2, IL-5, IL-17E and TSLP, with regard to the control and SAC groups, and IL-4, IL-13, IL-17F, IL-21 and IL-22, with regard only to the control group.

Finally, IL-6, IL-10, IL-12p70, IL-17A, RANTES and TNF-α concentrations in PAC and IgA and MMP-9 concentrations in SAC were increased when comparing the allergic groups.

#### Analysis of Lower Bulbar Conjunctival Microbiota

**Figure 2:**
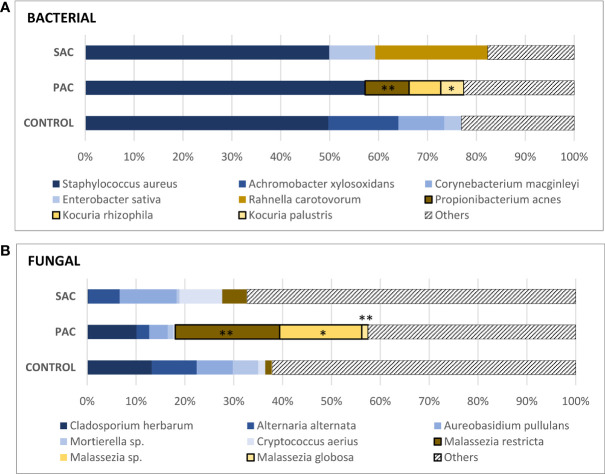
Mean values of the most common species of bacteria **(A)** and fungi **(B)**. *Propionibacterium acnes* (PAC: 9.01 ± 9.93%), *Kocuria rhizophila* (PAC: 6.49 ± 19.46%)*, K. palustris* (PAC: 4.71 ± 9.26%). *Malassezia restricta* (SAC: 5.01 ± 12.19%, PAC: 21.71 ± 18.54%, C: 1.28 ± 2.03%), *Malassezia species.* (PAC: 17.07 ± 24.29%), Malassezia globosa (PAC: 1.33 ± 1.54%). *“Malassezia species”* refers to fungi of the genus *Malassezia* whose species have not been identified. The Kruskal-Wallis test was applied to compare the control, PAC and SAC groups. *p < 0.05, **p < 0.01.

**Figure 3:**
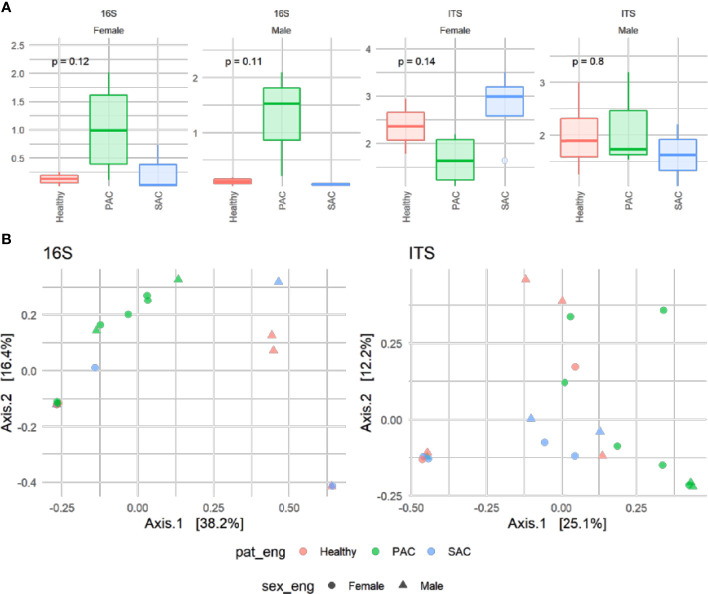
Alpha diversity determined with Shannon index for prokaryotic (16S) and fungal communities (ITS) subdivided by sex and categorized by pathology. Resulting p-values of Kruskall-Wallis are shown on the top **(A)**. Principal Coordinate analysis (PCoA) of the microbiota based on Bray-Curtis distance matrix of patients annotated by sex (shape) and pathology (color) categories **(B)**.

For this part, 22 volunteers (seven control, nine PAC and six SAC) were studied. A total of 538 bacteria and 648 fungi species were analyzed by high-throughput DNA sequencing. The averages of all analyzed species were calculated for each group. Bacterial and fungal species with a detection frequency under 30% in any group were included as “others,” irrespective of their average. Results are summarized in **Figure 2**, while alpha diversity index comparison for both 16S and ITS markers did not show any significant difference among pathology, but PAC group tended to show higher diversity for both markers. Conversely, ordination of microbiome composition for both revealed that PAC samples seem to cluster apart from the rest of the pathology groups (**Figure 3**).

Regarding the bacterial microbiome, around 50% of the analyzed DNA belonged to *Staphylococcus aureus* in both the healthy and allergic groups. After *S. aureus*, other species, such as *Enterobacter sativa* and *Rahnella carotovorum*, had the highest percentages in SAC, although these two populations were only found in one-third of the samples. The same condition was observed for healthy individuals: *Acromobacter xylosoxidans*, *Corynebacterium macginleyi*, and *E. sativa* had high percentages but were only found in one-third of healthy samples. This is due to the high DNA percentages reached for these species in some patients.

Surprisingly, all PAC samples were positive for one of the *Kocuria* species (*rhizophila*: 6.49 ± 19.46%; *palustris*: 4.71 ± 9.26%) and *Propionibacterium acnes* (9.01 ± 9.93%).

Regarding fungal communities, something similar occurred, the DNA of *Malassezia* species was detected in all PAC samples, with the *restricta* variety being the most abundant (*M. restricta:* 21.71 ± 18.54%, *Malassezia* species: 17.07 ± 24.29%, and *M. globosa:* 1.33 ± 1.54%). Together, the three *Malassezia* populations comprised 40.05% of PAC microbiota. Other fungal populations, such as *Cladosporium herbarum*, *Alternaria alternata*, *Aureobasidium pullulans*, *Mortierella* species or *Cryptococcus aerius* were present in both healthy and allergic individuals in different proportions.

## Discussion

It goes without saying that the CALT-tear-microbiota triad will play an important role in the immune response and ocular surface protection. Our study provided a detailed view of the ocular surface, improving knowledge of conjunctival IEL phenotypes, lacrimal soluble molecules and commensal microbial communities in both healthy individuals and allergic patients.

### A Detailed Model of Healthy Conjunctival IELs

As can be seen in **Table 3**, lymphocytes with innate immune function (MAIT, CD8+ T cells, NKT, CD3+TCRγδ+, and B1) have higher proportions in the conjunctiva when compared with peripheral blood lymphocytes. This suggests that the ocular response against pathogens will be mainly a strong, fast and non-specific response.

With the exception of the higher proportions of CD8+ T cells, which is a common mucosal feature previously observed in the conjunctiva (4), the remaining findings are completely new in this tissue.

According to many authors, CD8 T lymphocytes, as well as MAIT and NKT cells, play an important role in mucosal defense. They are characterized by their great versatility, from a cytotoxic function, generating a wide variety of cytokines (IFN-γ, IL-4, IL-13, IL-17 and IL-22) to immunoregulatory functions, serving as a bridge between innate and adaptive immunity (15–17).

Complementing this innate immunity, a CD4+ sentinel population exists with a strong effector function, ready for possible infections. Our immunophenotypic analysis demonstrated that about two-thirds of CD4 T cells were activated (CD45R0+) in the conjunctiva, being approximately 50% Treg cells. These findings suggest that the conjunctiva have a greater regulatory capacity against a possible uncontrolled Th cell activity. In fact, our results also revealed that Treg proportions were constant regardless of allergic conjunctivitis, while the number of Th subsets could vary with the development of the allergic process.

**Figure 4:**
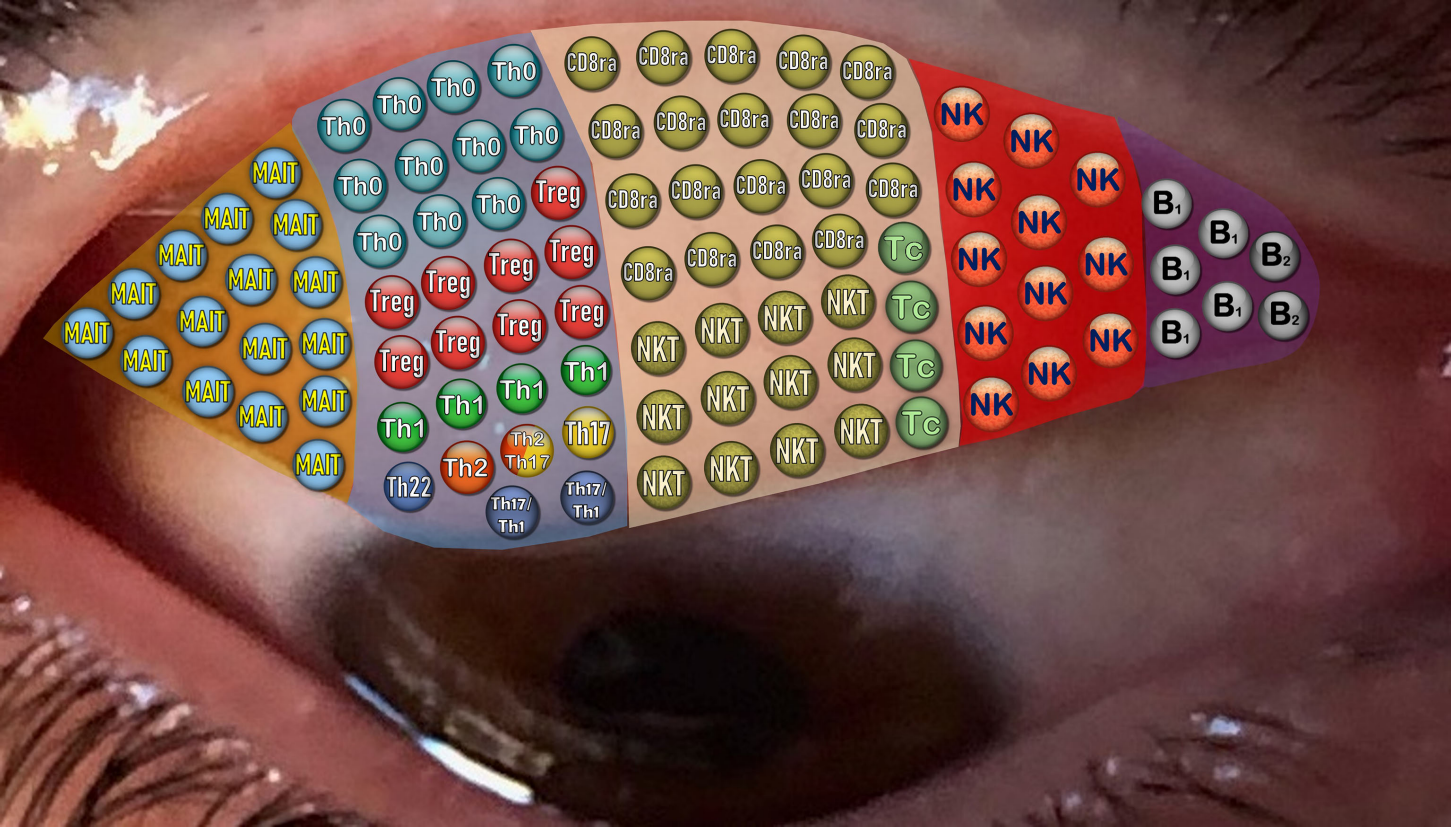
Picture showing lymphoid subpopulation proportions in healthy conjunctiva using spheres. Different colored regions represent MAIT, CD4 T, CD8 T, NK and B cells, randomly observed in the conjunctiva. As can be seen, there is a fairly abundant mucosal associated invariant T (MAIT) cell population expressing CD3+ but not CD8 and CD4. Note that T cells are predominantly CD8+, being almost 50% CD8 memory T cells (CD45r0), with a NKT cell predominance. In contrast, CD4 lymphocytes are mainly memory T cells, with a Treg predominance. Finally, B1 cells predominate over B2 cells in the CD19+ group.

Using the obtained values, we can get an overview of the proportions of each IEL subpopulation in healthy individuals, as summarized in **Figure 4**.

### IELs and Tears in the Allergic Process

Once the allergic process starts, CALT becomes active, changing its phenotype and increasing epithelial infiltration. Then, IELs and other immune system cells, goblet cells and epithelial cells, among others, will liberate pro-inflammatory molecules into the tear fluid, producing conjunctival inflammation.

In addition to innate immunity lymphocytes, such as NKTs, T helper cells play an important role in allergic diseases. Our results reveal a T helper polarization to pro-inflammatory phenotype (Th2, Th17 and Th22) to the detriment of the immunomodulatory phenotype (Th1 and Th17/Th1) in allergic conjunctivitis. These lymphoid variations have already been demonstrated in ocular allergy regarding the lacrimal cytokine profile (3, 5), but we have not found studies analyzing conjunctival IELs and T helper cell subpopulations.

According to results, we can conclude that there is a clear difference between healthy and allergic subjects in conjunctival IELs. PAC is characterized by overexpression of Th2 and NKT cells, with a decrease of the TCRγδ and MAIT subsets. However, SAC is characterized by a marked decrease in Th1 cells with an increase in the Th17 and Th22 subsets.

Our data for NKT, TCRγδ and MAIT cells in the conjunctiva are totally novel, and no reference data from other studies could be found. In recent years, these three lymphocyte populations have gained prominence in mucosal defense. A mouse model of allergic conjunctivitis showed that NKT cells were necessary for maximum expression of allergic conjunctivitis (9), suggesting that NKT cells play an important role in the development of disease. The same applies to MAIT and TCRγδ+ lymphocytes, considered as the communication bridge between commensal microbiota and the mucosal-associated immune system (18, 19).

Th2 cells are closely related to allergic reactions, as they are responsible for activating B cells and initiating the IgE-mediated response. In contrast, the role of Th17 cells in conjunctivitis is less well understood. Th17 cells are commonly known to play a pro-inflammatory role, and it is thought that they may aggravate the Th2 cell inflammatory response in allergic conjunctivitis (20).

Moreover, Th22 cells have a dual role by participating in the allergic inflammatory process and promoting epithelial regeneration (21). These cells have been shown to be related to skin diseases and may be increased in atopy (dermatitis and psoriasis) (22).

Concerning the tear component, we have found a predominantly Th2 environment in both healthy and allergic patients, led by IL-4 and IL-13, followed by a Th17-type response, led by IL-17F and IL-22. These four cytokines represent between 50-60% of the interleukins in tears.

We have also seen that both PAC and SAC are characterized by an increased IgE and decreased TGF-β2 isotype. An increase in IgE concentration is commonly seen in the tears of allergic conjunctivitis patients (23). In contrast, the decreased TGF-β2 is somewhat less studied, although the same decrease has been observed in dry eyes (24).

Furthermore, we can generally say that perennial conjunctivitis is characterized by an increase in some pro-inflammatory molecules, such as Th1 (IFN-γ and IL-2), Th2 (IL-4, IL-5 and IL-13) and Th17 (IL-17A, IL-17E, IL-17F, IL-21 and IL-22), as well as IL-β1 and TSLP, when compared to healthy individuals. Several studies have associated an increase in certain tear soluble molecule concentrations with the development of various inflammatory diseases. Increases of IFN-γ, IL-2, IL-4, IL-5, IL-13 or IL-β1 in SAC (25); TSLP in both PAC and SAC (26); and IL-17, IL-21 and IL-22 in dry eyes (27, 28) have been observed in different studies.

In return, seasonal conjunctivitis differs from perennial conjunctivitis with regard to higher concentrations of IgA and MMP-9. Some studies have reported an increase in IgA concentrations in acute forms of different forms of conjunctivitis (29, 30). However, other recent studies have reported a decrease (31). Regarding MMP-9, some studies have reported an increased concentration in vernal keratoconjunctivitis tears (32) and dry eyes (33).

The tear molecular profile in seasonal conjunctivitis is far from expected, considering previous studies that have analyzed tears in these ocular allergies. As observed in other studies, cytokine concentrations were expected to be similar to, or even higher than, those obtained for perennial conjunctivitis. This could mean that patients with SAC were in a post-inflammatory or recovery stage, which would explain the reduced symptomatology at the time of examination, despite the presence of conjunctival inflammation.

Looking at the IEL values in SAC, we would expect to find an increase in the Th17 and Th22 cytokine concentrations in tears, but instead, we only found a significant increase in IgA, IgE and MMP-9.

However, in PAC, we found a concordance between Th2 increase in the conjunctiva and IL-4, IL-5 and IL-13 increase in the tears. Therefore, we associated the other increased cytokines, such as IL-17, IL-22 and IFN-γ, with the NKT cell increase and its varied cytokine repertoire.

### The Microbiota’s Contribution to Allergic Conjunctivitis

Our data show some interesting differences in the allergic conjunctivitis microbiome. Both bacterial and fungal colonies show that the PAC group has a different microbiota compared to the SAC and control groups. Interestingly, *Kocuria*, *P. acnes* and *Malassezia* species DNA was detected in all the samples from the PAC group, but not in the other groups. It should be added that microbiota samples were collected during 2018 and 2019 in the spring and summer months. The sample collection dates were reviewed, and no significant differences were observed from samples collected in different seasons or years. It suggests that group differences are purely allergy-driven, providing further strength to our results and demonstrating a direct association between commensal microbiota, development of perennial conjunctivitis and its symptomatology.

Numerous studies have shown a close relationship of dysbiosis and development of allergic disease in different anatomical areas (12). In particular, several studies have related skin diseases, such as psoriasis and atopic dermatitis, to abundances of certain fungal and bacterial species, including *Kocurias* and *Malassezia* (34–36).

Furthermore, it is well known that there is cross-regulation involving mucosal lymphocytes and commensal microbiota in a variety of inflammatory diseases. For example, commensal microbiota play an important role in the regulation of NKT cells in the lungs and intestines (37). Other studies have shown that microbiota also regulate the Th17/Treg balance in the lamina propria of intestinal mucosa, affecting the development of inflammatory disease (38).

Combining findings on the conjunctiva, tear and commensal microbiota, it seems that abundant mucosal colonization by specific bacterial or fungal species will produce an imbalance in ocular surface homeostasis and, consequently, a pronounced inflammatory response. An example of this is clearly seen in perennial conjunctivitis, where *Kocuria*, *P. acnes* and *Malassezia* species are overpopulated. Indeed, this growth may be related to T helper cell polarization to Th2 and CD8 T cells to NKT cells, resulting in increased production of Th2- and Th17-type cytokines.

On the other hand, it is also probable that the reduction of the TCRγδ and MAIT cells in the PAC group is associated with the dysbiosis observed. However, we do not yet know whether the cause or effect is, if this cell reduction, which could be physiological, allows the colonization of other microbial species, or this microbial overpopulation is the reason for this lymphoid reduction.

Additionally, we have no knowledge about the origin of these “invasive” species. The colonies observed in PAC (*Kocuria*, *Malassezia*, and *P. acnes*) are certainly species commonly found on human mucous membranes and skin, but they can also be located in soils, animals or the environment (39, 40). For this reason, we propose two possible theories: an endogenous theory and an exogenous theory.

Different studies have shown that these species are related to skin diseases such as dermatitis (41, 42), where over-colonization of these microbial species has been found, especially in areas where there are numerous sebaceous glands (as occurs in the eyelids) (43). Within the allergic groups, a considerable number of subjects reported having suffered from dermatitis at some point in their lives, so it is not unreasonable to think that they continued to have active skin disorders or blepharitis, making the ocular surface an appropriate place for the expansion of these species.

The other theory, less probable in our opinion, is the existence of a certain seasonality. These colonies, in particular *kocuria* species, could be found in the environment during harvesting and high pollen concentration periods. But in that case, we would have found these species also in the rest of the groups studied. Therefore, it is possible that in the control and SAC groups, where MAIT and TCRγδ values were not decreased, the invasive species could have been eliminated while the deficit of lymphocytes in PAC allowed dysbiosis.

It seems clear that a different microbial profile exists in PAC, distinctive for this allergic conjunctivitis, which could play an important role in the allergy development, prevalence, and symptomatology. However, further research is needed to answer all the emerging questions. For example, sampling ocular microbiota in different seasons to confirm the absence of seasonality, while a microbiological study of the environment is performed, sampling microbiota in other patient areas looking for a similar profile or measuring the neutrophil infiltration and their potential role in the ocular microbiota regulation.

In summary, we found several interesting differences in allergic conjunctivitis groups, especially in PAC. If we consider that patients with SAC are in a post-allergic or recovery stage and patients with PAC are in a high stage of allergic development, the results look more interesting and make more sense. The early phase will have high levels of Th1, MAIT and TCRγδ cells. These cells, along with other innate cells, are responsible for initiating the pro-inflammatory response. As the response matures, these cells are reduced, allowing the growth of Th2 and NKT cells and the release large amounts of cytokines into the tears. When the allergic stimulus ends, the ocular surface goes into a recovery phase. This phase, as we have observed in SAC, is characterized by an increase in Th17 and Th22 cells; IgA; and MMP-9, while the proportions of NKT, MAIT and TCRγδ cells; IgE; and TGF-β2 gradually return to baseline values. Th17, Th22 and MMP-9 are responsible for epithelial regeneration and together with IgA promote the return to mucosal homeostasis.

Therefore, our findings suggest that the activation of the allergic process relies initially on MAIT and TCRγδ+ cells, as well as, in some still unknown way, the commensal microbiota. Th2 and NKT cells show their highest expression during the later phases.

The strong differences found in conjunctival IELs, tear molecules and microbial species in PAC leads us to propose these facts as differential PAC biomarkers as follows:

1. A higher number of Th2 vs Th1 cells, combined with an increase in NKT cells and a decrease in TCRγδ+ and MAIT cells, in the conjunctiva.
2. High concentrations of IgE and Th2 or Th17 profile cytokines coupled with low TGF-β2 concentrations in tear fluid.
3. An overpopulation of *Kocuria*, *P. acnes* or *Malassezia* species in ocular microbiota.

This study opens the door to further investigations in order to introduce new therapeutic and diagnostic strategies for allergic conjunctivitis and clarifies who the main players are in the allergic inflammatory process.

## Data Availability Statement

The original contributions presented in the study are publicly available. This data can be found here: https://www.ncbi.nlm.nih.gov/bioproject/PRJNA831644/.

## Ethics Statement

The studies involving human participants were reviewed and approved by by the institutional review board and by the Ethics Commitee of the Valladolid East (ref.: PI -14-213) and West (ref.: PI -14-213) Health Area. Written informed consent to participate in this study was provided by the participants’ legal guardian/next of kin.

## Author Contributions

AC and RR contributed to conception and design of the study. JZ wrote the first draft and sections of the manuscript. AA, AC and RR contributed to the recruitment of volunteers. AA supervised the testing of volunteers. AE supervised and assisted in the preparation of all cytokine analysis. JZ organised patient meetings, performed all patient examinations, and extracted the cytological, tear and microbiota samples. JZ performed all laboratory techniques and analysis of results. All authors contributed to the article and approved the submitted version.

## Funding

This study was co-financed by the Spanish Ministry of Economy and Competitiveness, (Government of Spain), the Institute of Health Carlos III (ISCIII) and the European Regional Development Fund (Grant number PI14/01886, “INFLAMMATORY BIOMARKERS FOR DIFFERENTIAL DIAGNOSIS AND MONITORING OF ALLERGIC CONJUNCTIVITIS", University of Valladolid, Spain).

## Conflict of Interest

The authors declare that the research was conducted in the absence of any commercial or financial relationships that could be construed as a potential conflict of interest.

## Publisher’s Note

All claims expressed in this article are solely those of the authors and do not necessarily represent those of their affiliated organizations, or those of the publisher, the editors and the reviewers. Any product that may be evaluated in this article, or claim that may be made by its manufacturer, is not guaranteed or endorsed by the publisher.
