# Supplementary material for: Conjunctival Intraepithelial Lymphocytes, Lacrimal Cytokines and Ocular Commensal Microbiota: Analysis of the Three Main Players in Allergic Conjunctivitis
Source: Front Immunol. 2022 Jul 19;13:911022. doi: 10.3389/fimmu.2022.911022 (PMC9351602; doi:10.3389/fimmu.2022.911022)
Supplement: Supplementary file 1 [file DataSheet_1.pdf]

Supplementary Material

Supplementary Figure 1. Gating strategies used to analyze conjunctival IELs and blood lymphocytes. The samples were divided into 5 tubes and analyzed in 5 panels as shown below. (A) Panel 1 - Naïve and memory T subsets. (B) Panel 2 - T helper subsets. (C) Panel 3 - Tregs Lymphocytes. (D) Panel 4 - NK and NKT subpopulations. (E) Panel 5 - TCR cells and B subpopulations

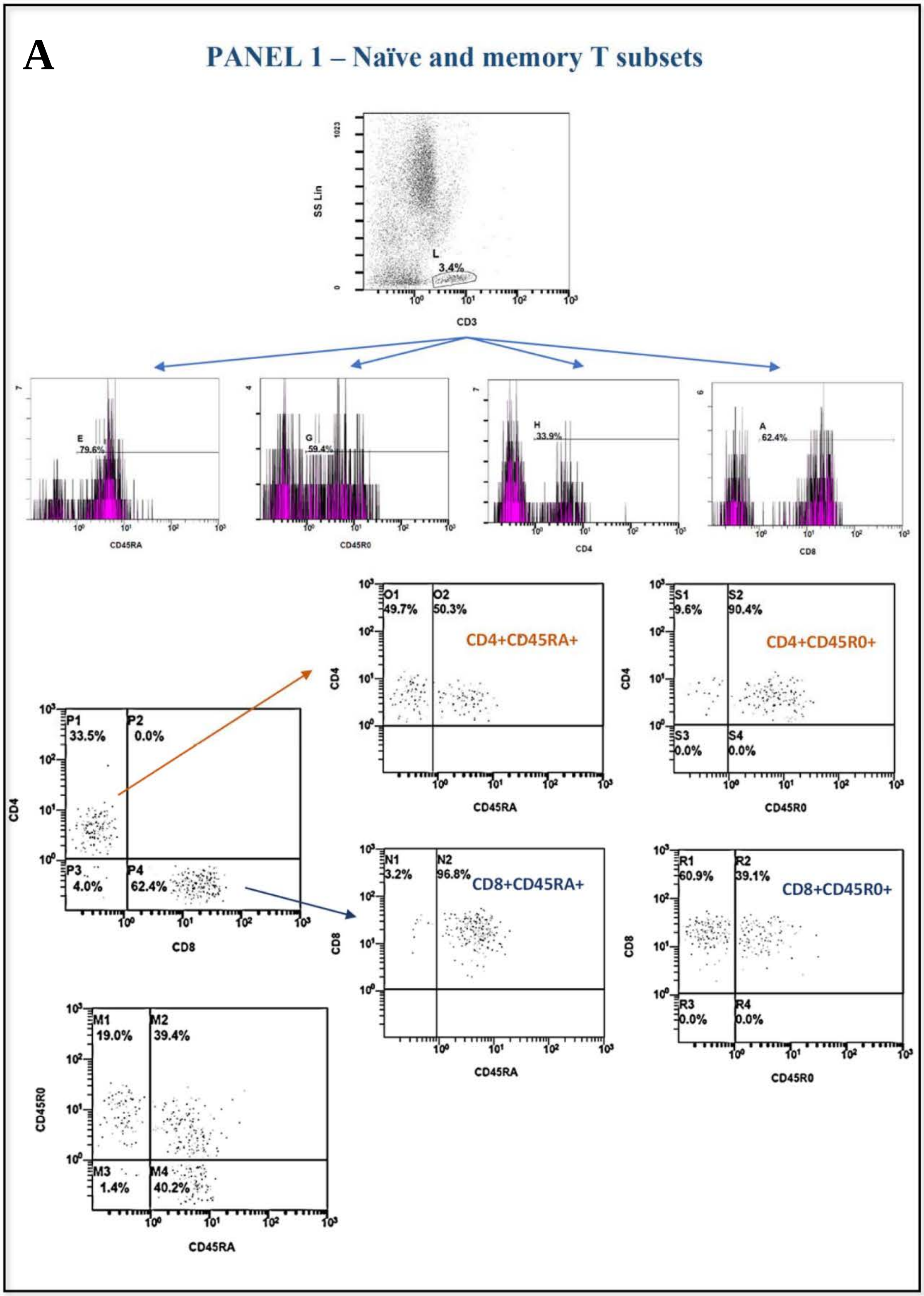
A
PANEL 1 – Naïve and memory T subsets
SS Lin
CD3
L
3.4%
E
79.6%
CD45RA
G
59.4%
CD45R0
H
33.9%
CD4
A
62.4%
CD8
P1
33.5%
P2
0.0%
P3
4.0%
P4
62.4%
O1
49.7%
O2
50.3%
CD4+CD45RA+
S1
9.6%
S2
90.4%
S3
0.0%
S4
0.0%
CD4+CD45R0+
N1
3.2%
N2
96.8%
CD8+CD45RA+
R1
60.9%
R2
39.1%
R3
0.0%
R4
0.0%
CD8+CD45R0+
M1
19.0%
M2
39.4%
M3
1.4%
M4
40.2%

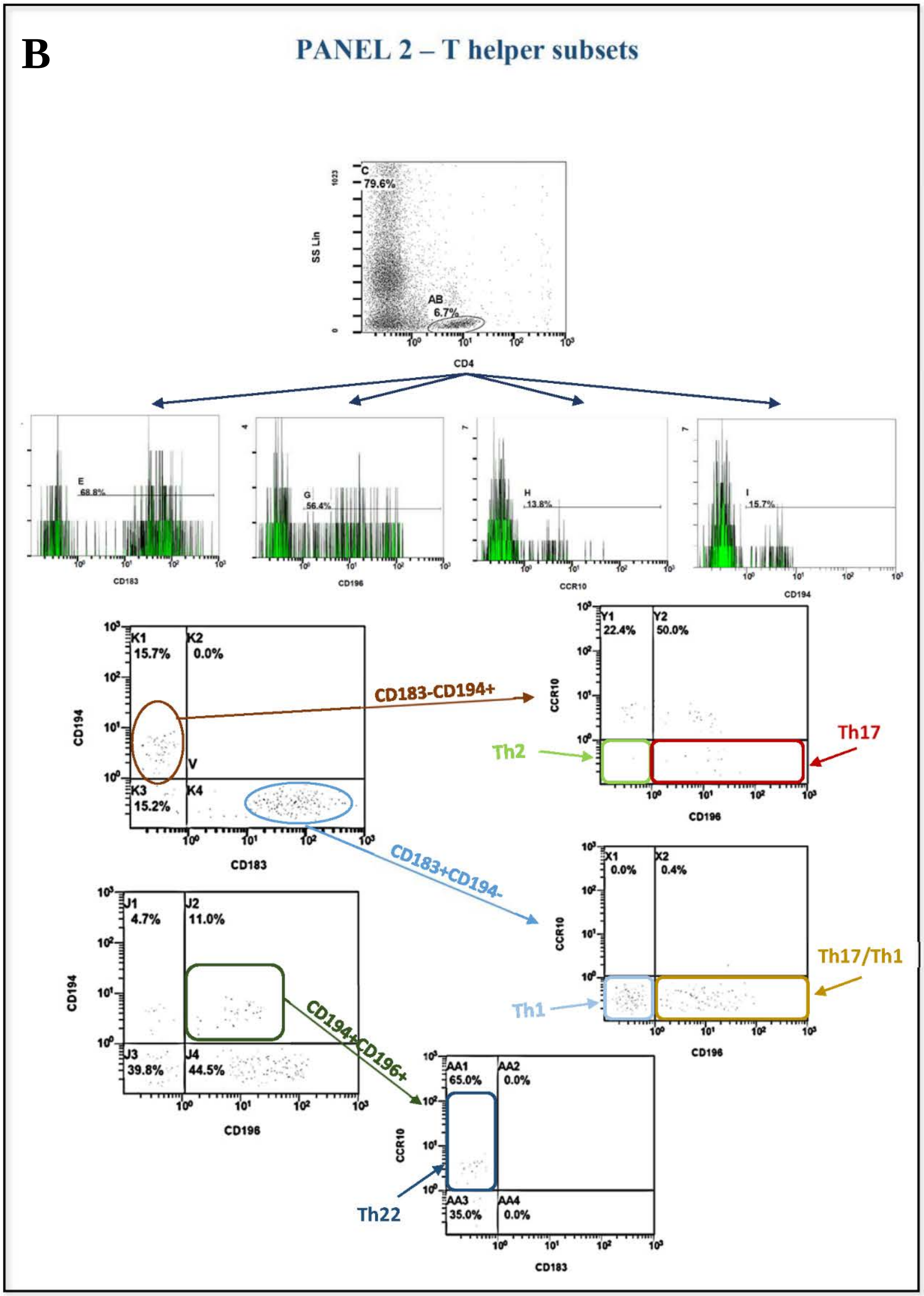
B
PANEL 2 – T helper subsets
C
79.6%
SS Lin
AB
6.7%
CD4
E
68.8%
CD183
G
56.4%
CD196
H
13.8%
CCR10
I
15.7%
CD194
K1
15.7%
K2
0.0%
K3
15.2%
K4
CD194
CD183
CD183-CD194+
CD183+CD194-
Y1
22.4%
Y2
50.0%
CCR10
CD196
Th2
Th17
X1
0.0%
X2
0.4%
Th17/Th1
Th1
J1
4.7%
J2
11.0%
J3
39.8%
J4
44.5%
CD196
CD194+CD196+
AA1
65.0%
AA2
0.0%
AA3
35.0%
AA4
0.0%
CD183
Th22

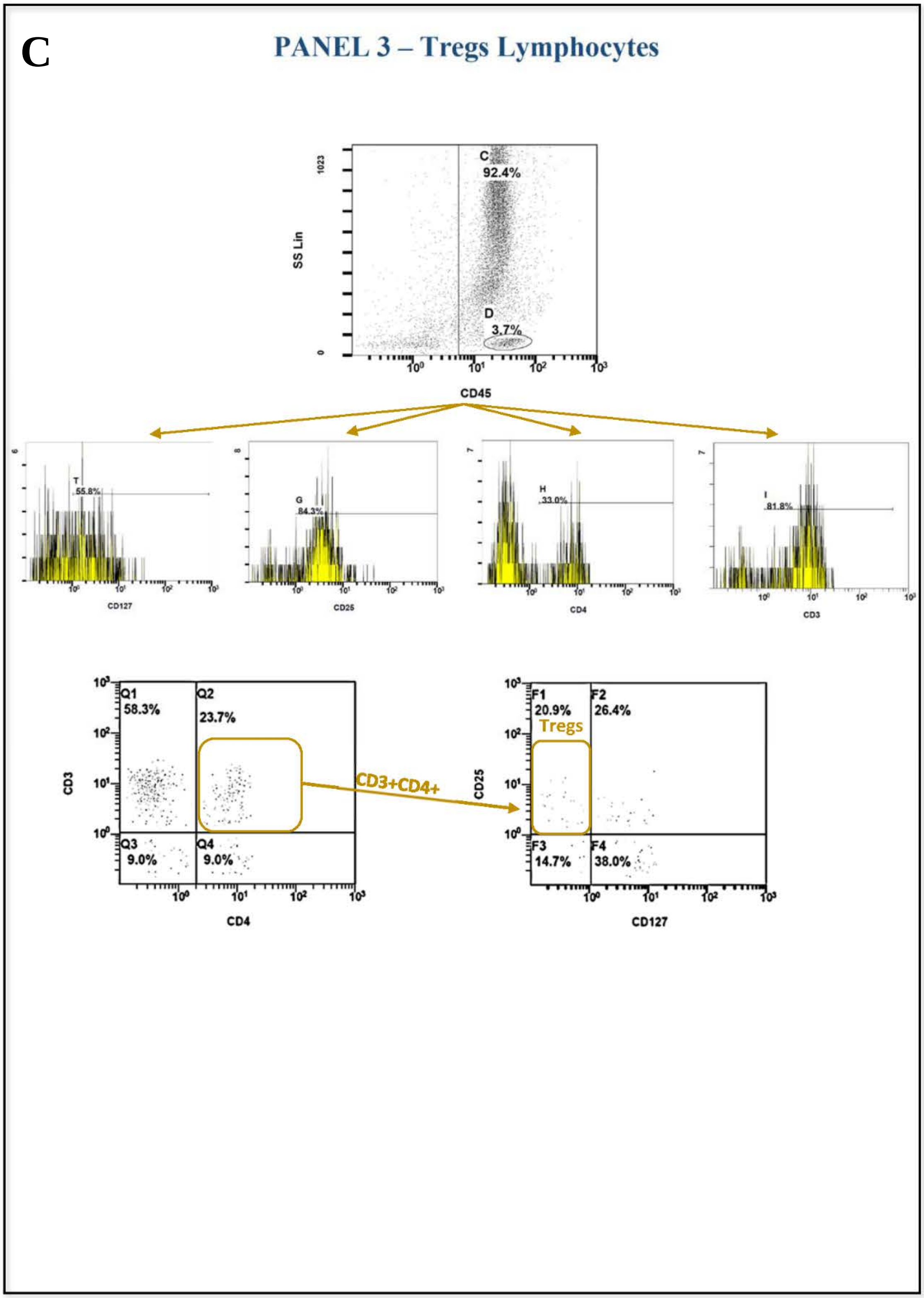
C
PANEL 3 – Tregs Lymphocytes
C
92.4%
D
3.7%
SS Lin
CD45
T
55.8%
CD127
G
84.3%
CD25
H
33.0%
CD4
I
81.8%
CD3
Q1
58.3%
Q2
23.7%
Q3
9.0%
Q4
9.0%
CD3
CD4
CD3+CD4+
F1
20.9%
Tregs
F2
26.4%
F3
14.7%
F4
38.0%
CD25
CD127

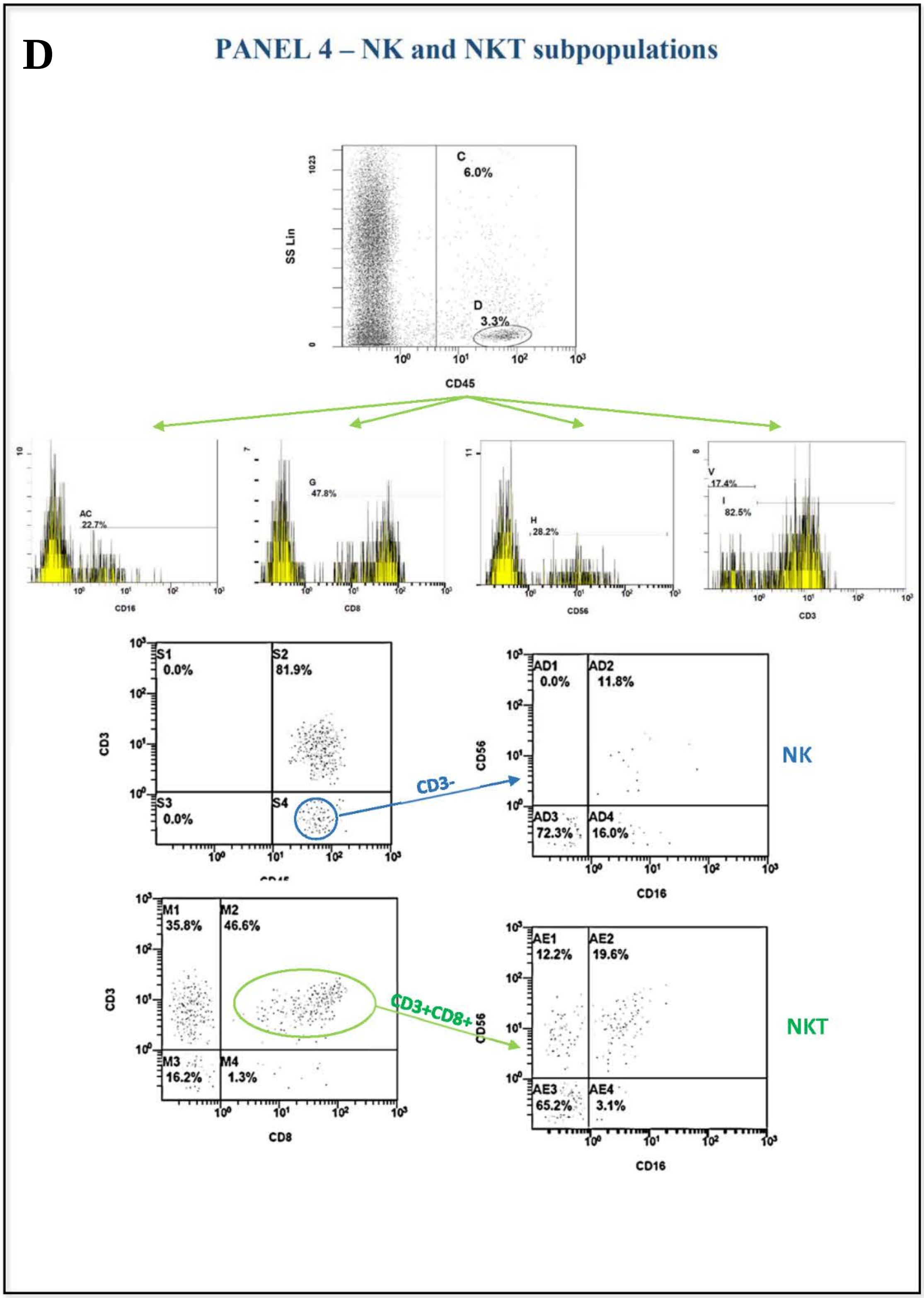
D
PANEL 4 – NK and NKT subpopulations
SS Lin
CD45
C
6.0%
D
3.3%
AC
22.7%
CD16
G
47.8%
CD8
H
28.2%
CD56
V
17.4%
I
82.5%
CD3
S1
0.0%
S2
81.9%
S3
0.0%
S4
CD3-
AD1
0.0%
AD2
11.8%
AD3
72.3%
AD4
16.0%
NK
M1
35.8%
M2
46.6%
M3
16.2%
M4
1.3%
CD3+CD8+
AE1
12.2%
AE2
19.6%
AE3
65.2%
AE4
3.1%
NKT

E

PANEL 5 – TCRγδ+ cells and B subpopulations

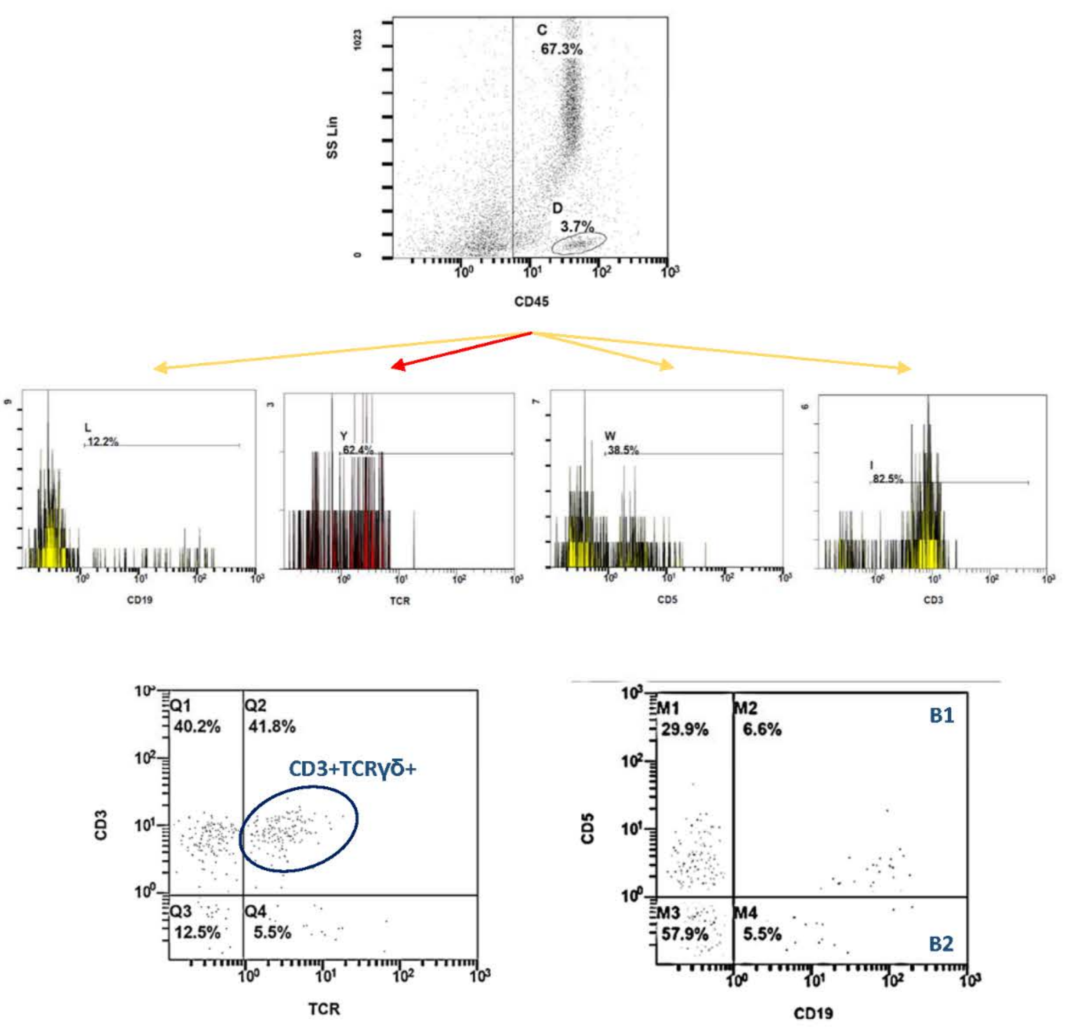

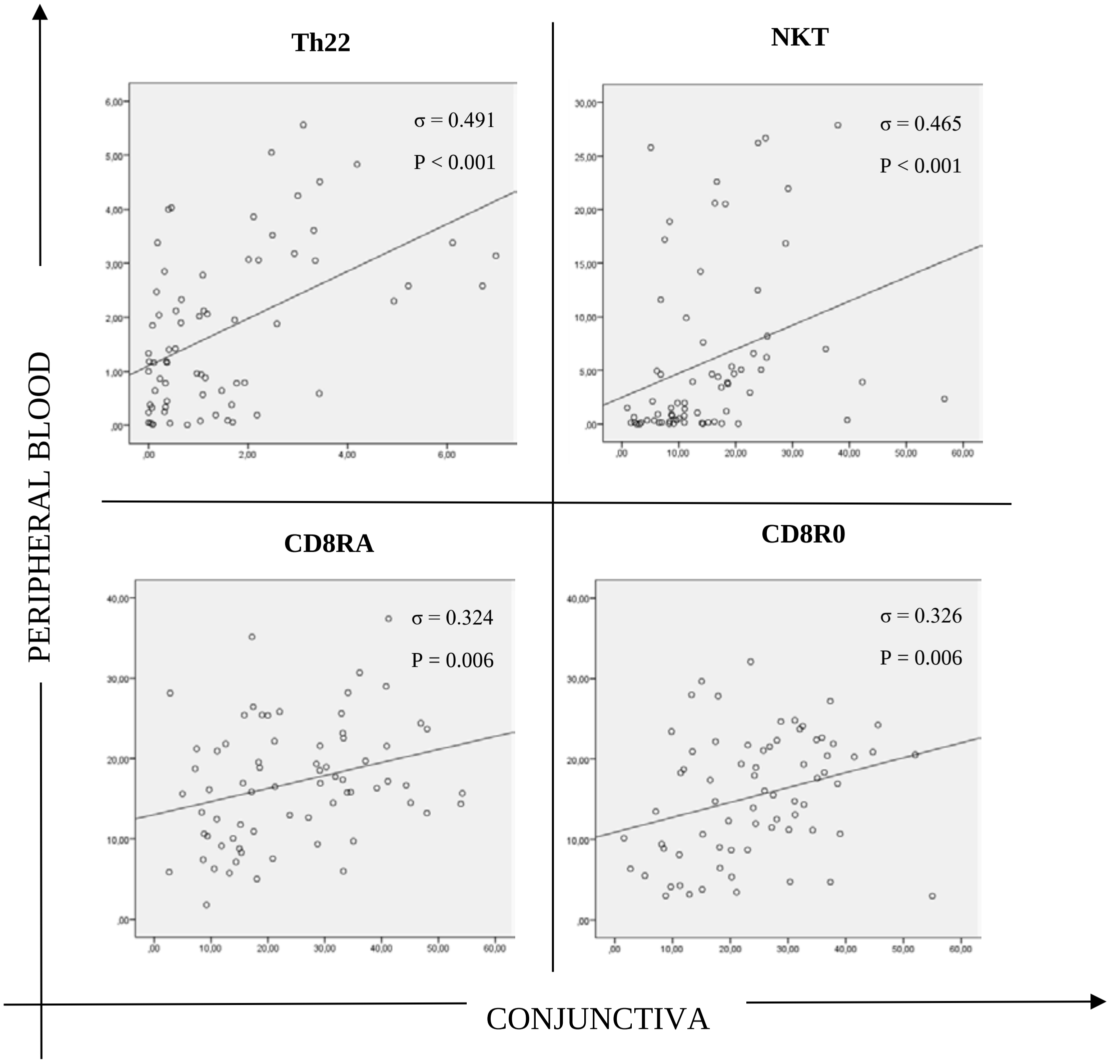

Supplementary Figure 2. Scatter plots showing positive correlations for Th22, NKT, CD8RA and CD8R0. σ = Spearman's σ correlation coefficient. σ in the range of 0.3 to 0.6 were considered as moderate correlation.
